# Toll-Like Receptor Agonists Modulate Wound Regeneration in Airway Epithelial Cells

**DOI:** 10.3390/ijms19082456

**Published:** 2018-08-20

**Authors:** Anna Lewandowska-Polak, Małgorzata Brauncajs, Marzanna Jarzębska, Małgorzata Pawełczyk, Marcin Kurowski, Maciej Chałubiński, Joanna Makowska, Marek L. Kowalski

**Affiliations:** 1 Department of Rheumatology, Chair of Clinical Immunology and Rheumatology, Medical University of Lodz, 90-419 Lodz, Poland; joanna.makowska@umed.lodz.pl; 2 Department of Microbiology and Medical Laboratory Immunology, Medical University of Lodz, 90-419 Lodz, Poland; malgorzata.brauncajs@umed.lodz.pl; 3 Department of Immunology and Allergy, Chair of Clinical Immunology and Rheumatology, Medical University of Lodz, 90-419 Lodz, Poland; marzanna.jarzebska@umed.lodz.pl (M.J.); malgorzata.ewa.pawelczyk@umed.lodz.pl (M.P.); marcin.kurowski@umed.lodz.pl (M.K.); maciej.chalubinski@umed.lodz.pl (M.C.); marek.kowalski@umed.lodz.pl (M.L.K.)

**Keywords:** airway epithelium, wound repair, TLRs, poly(I:C), LPS

## Abstract

Background: Impaired regeneration of airway epithelium may lead to persistence of inflammation and remodelling. Regeneration of injured epithelium is a complex phenomenon and the role of toll-like receptors (TLRs) in the stimulation of respiratory virus products in this process has not been established. Objective: This study was undertaken to test the hypothesis that the wound repair process in airway epithelium is modulated by microbial products via toll-like receptors. Methods: Injured and not-injured bronchial epithelial cells (ECs) (BEAS-2B line) were incubated with the TLR agonists poly(I:C), lipopolisacharide (LPS), allergen Der p1, and supernatants from virus-infected epithelial cells, either alone or in combination with TLR inhibitors. Regeneration and immune response in injured and not-injured cells were studied. Results: Addition of either poly(I:C) or LPS to ECs induced a marked inhibition of wound repair. Supernatants from RV1b-infected cells also decreased regeneration. Preincubation of injured and not-injured ECs with TLR inhibitors decreased LPS and poly(I:C)-induced repair inhibition. TGF-β and RANTES mRNA expression was higher in injured ECs and IFN-α, IFN-β, IL-8, and VEGF mRNA expression was lower in damaged epithelium as compared to not-injured. Stimulation with poly(I:C) increased IFN-α and IFN-β mRNA expression in injured cells, and LPS stimulation decreased interferons mRNA expression both in not-injured and injured ECs. Conclusion: Regeneration of the airway epithelium is modulated by microbial products via toll-like receptors.

## 1. Introduction

The airway epithelium plays a protective role as a physical and functional barrier between the external milieu and underlying tissues. This barrier is constantly injured and epithelial integrity is damaged. Different factors, such as allergens, microbes, tobacco smoke, and airborne particulates, may induce injuries of airway epithelium, requiring a regulated repair mechanism to restore the protective barrier [1,2]. A wound repair process starts quickly after injury and includes activation and migration of cells from the epithelial basal layer to repopulate the damaged area followed by cell proliferation and differentiation as well as changes in gene expression. This process provides the possibility for complete regeneration of a pseudostratified mucociliary epithelium [1,3].

There is evidence that in airway inflammatory diseases, such as asthma and chronic rhinosinusitis, the barrier function of the airway epithelium is impaired [4,5]. In the past, most research has focused on investigating the allergic pathways of asthma, rhinitis, and rhinosinusitis, which are disorders frequently dominated by Th2 lymphocytes and eosinophilic inflammation in the airway [6]. However, allergy does not explain the origin of inflammation in non-allergic subjects. Moreover, structural changes of a remodeling nature and severity of inflammation are conditioned by the severity of asthma and not by the presence or absence of atopy or duration of symptoms [7]. According to further concepts, impaired repair responses in epithelium lead to persistence of inflammation and airway wall remodeling in asthma [8,9]. Therefore, the identification of factors, such as pathogens, that may modulate airway epithelial repair response and understanding cellular and molecular mechanisms that translate infection to epithelial cell injury and repair are of considerable clinical interest.

Viral infections are one of the main causes of asthma exacerbations and it has been shown that the presence of respiratory viruses, such as respiratory syncytial virus (RSV) or rhinovirus (RV) in the airway, and infection by these viruses cause epithelial tight junctions disruption and increased epithelial permeability [10,11,12,13]. Sajjan et al. showed that human rhinovirus disrupts the epithelial barrier and modulates innate immunity defence mechanisms [10]. However, there are only a few studies that have shown the effect of rhinovirus on airway epithelial cell repair [14,15]. Bossios et al. reported that human rhinovirus induced cytotoxicity and impair wound healing in an in vitro model of tissue injury using immortalized human bronchial epithelial cell line-BEAS-2B. Faris et al. demonstrated that in this study rhinovirus reduced ciliated cell differentiation and promoted goblet-cell differentiation and mucin gene expression [14].

Epithelial barrier interacts with pathogens, such as bacteria and viruses, via toll-like receptors (TLRs), which are a group of molecules with a highly conserved structure, and their role is to recognize pathogens and to coordinate defense mechanisms against these pathogens. The impact of microbiota on the structural barrier’s integrity has been investigated in intestinal epithelium [16,17,18,19], but the role of pathogen recognition receptor (PRR) stimulation in airway epithelium regeneration is not well-established. There are only a few studies showing that microbial patterns signaling via Toll-like receptors contributes to epithelial repair, growth, and survival in the airway epithelium [20,21].

Impaired regeneration of epithelial cells may lead to persistence of inflammation and airway wall remodeling. Regeneration of injured epithelium is a complex phenomenon and the role of TLRs in the stimulation of respiratory virus products in this process is not well-established.

This study was undertaken to test the hypothesis that the wound repair process in airway epithelial cells is modulated by microbial products via toll-like receptors.

## 2. Results

### 2.1. Repair Response in Human Bronchial Epithelial Cells

**Figure 1:**
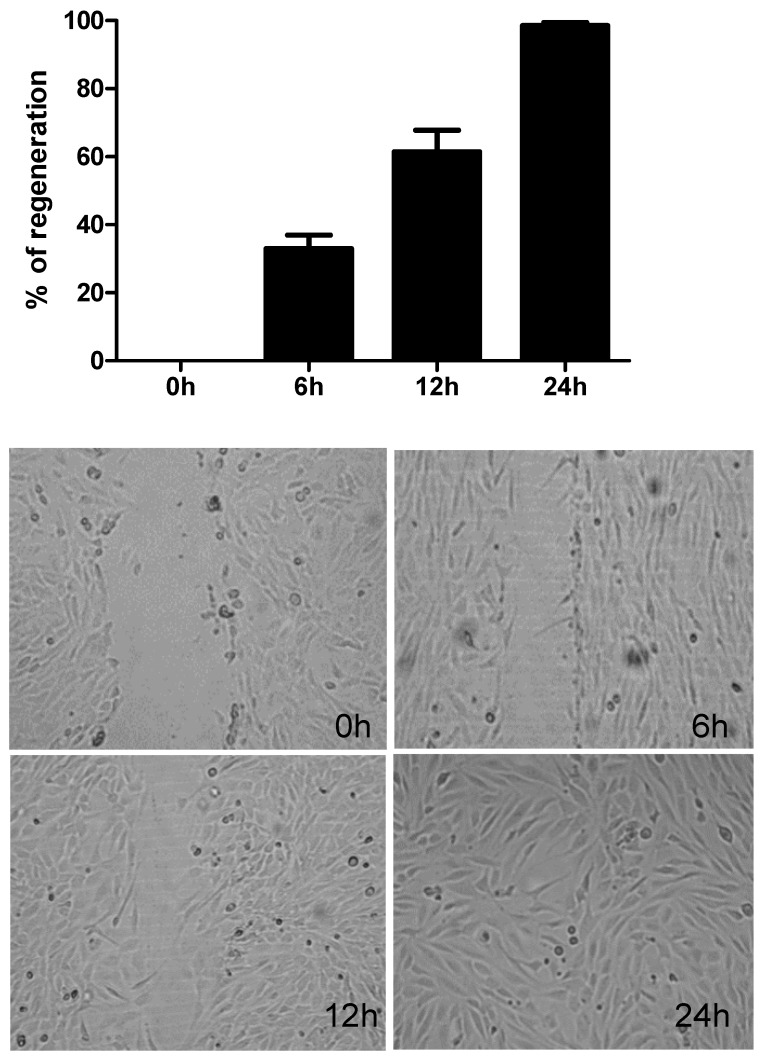
Wound repair in epithelial cells in response to mechanical injury. The magnification is 100×.

In preliminary experiments, we observed that in human bronchial epithelial cell cultures (BEAS-2B) a full closure of injury occurred from 18 to 26 h after injury (Figure 1). In the preliminary experiments, we have recorded the repair response every 3 h until full closure occurred. In the majority of cultures, full regeneration of damaged epithelium was observed 24 h post injury and in subsequent experiments we compared the effect of the TLR agonists Der p 1 and viral supernatants 24 h post injury.

### 2.2. Viability of Injured Immortalized Human Bronchial Epithelial Cells-BEAS-2B

Following injury, cell cultures were observed for 48 h under a light microscope and we did not observe detachment of cells and cell lysis. We were able to record cell proliferation and migration toward the injured surface.

When monolayers were stained with crystal violet, no disruption of the cell layer was observed 24 h and 48 h post injury. Viability of cells in injured cultures assessed with MTT did not differ from that in not-injured cultures.

### 2.3. Effect of TLR Agonists and Allergen on Wound Repair in Bronchial Epithelial Cells

**Figure 2:**
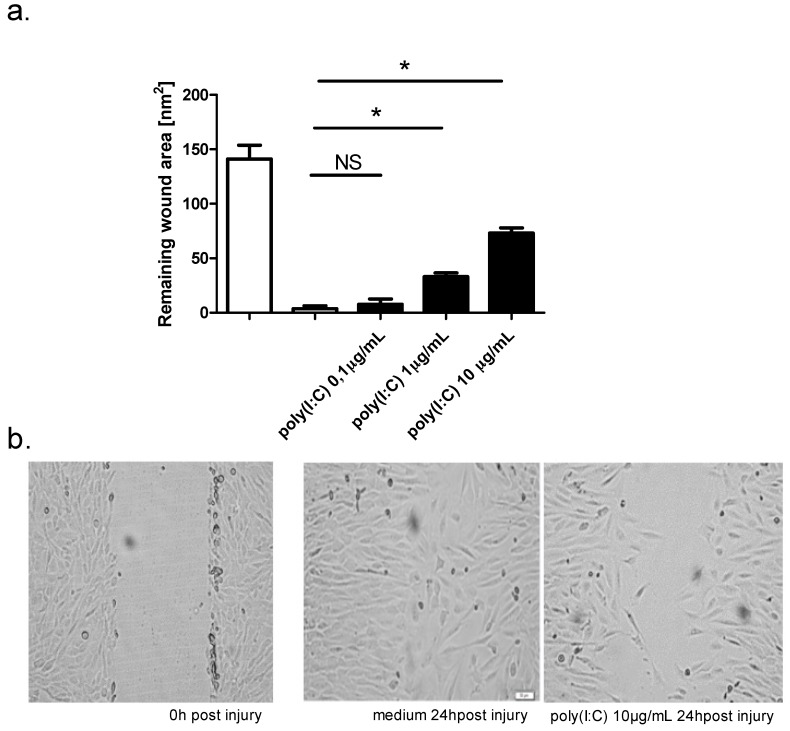
Effect of TLR agonist poly(I:C) on wound repair in BEAS-2B (*n* = 8), 24 h post injury. (**a**) Concentration dependent effect of poly(I:C) on wound repair in BEAS-B cultures, remaining wound area was measured 24 h post injury 2; (**b**) Representative images of injured BEAS-2B cells 0 h and 24 h post injury with or without poly(I:C) (10 μg/mL). NS, no statistically significant difference. The magnification is 100×; * *p* < 0.05.

After addition of poly(I:C) (0.1 μg/mL, 1 μg/mL, 10 μg/mL) to mechanically damaged monolayer cultures of BEAS-2B cells, a marked inhibition of wound repair was observed. The highest inhibition was observed for the highest concentration of poly(I:C) (10 μg/mL), and the lowest inhibition for 0.1 μg/mL (Figure 2a). Within 24 h, the control cultures had almost completely repaired and wound area measurements were done 12 h and 24 h post injury. Digital images of the control and poly(I:C)-treated cultures are shown in Figure 2b.

**Figure 3:**
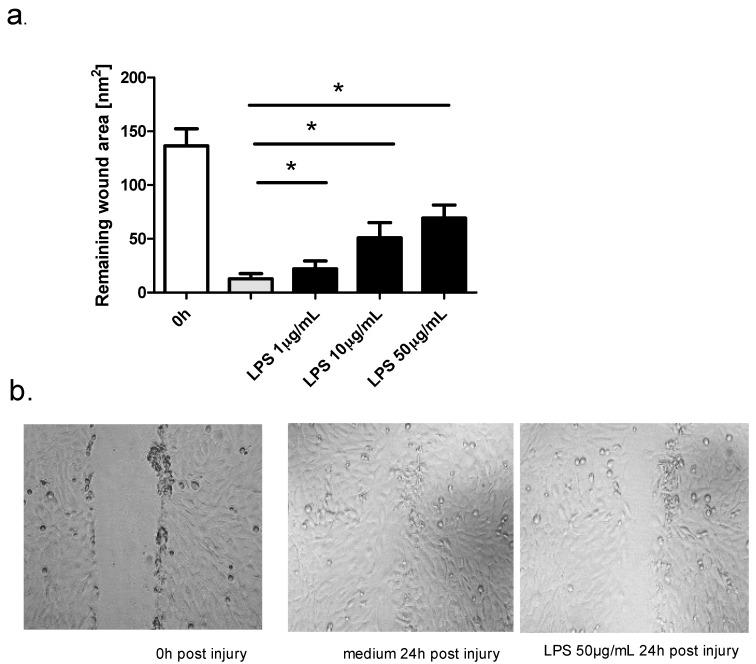
Effect of the toll-like receptor (TLR) agonist LPS on wound repair in BEAS-2B (*n* = 8), 24 h post injury. (**a**) Concentration dependent effect of LPS on wound repair in BEAS-B cultures, remaining wound area was measured 24 h post injury 2; (**b**) Representative images of injured BEAS-2B cells 0 h and 24 h post injury with or without LPS (50 μg/mL). NS, no statistically significant difference. The magnification is 100×; * *p* < 0.05.

The addition of LPS to damaged epithelial cells also resulted in decreased wound repair. We examined the effect of three concentrations of LPS (1 μg/mL, 10 μg/mL, and 50 μg/mL) on BEAS-2B repair, and the highest concentration of LPS caused the highest inhibition of epithelial regeneration (Figure 3a). Digital images of the control and LPS (50 μg/mL)-treated cultures are shown in Figure 3b. These data demonstrated that both LPS and poly(I:C) impair epithelial cells restitution in vitro. At higher concentrations of LPS (100 μg/mL), the decrease in wound repair was associated with a decrease in cell viability in the MTT assay. None of the three concentrations of poly (I:C) caused a decrease in cell viability.

**Figure 4:**
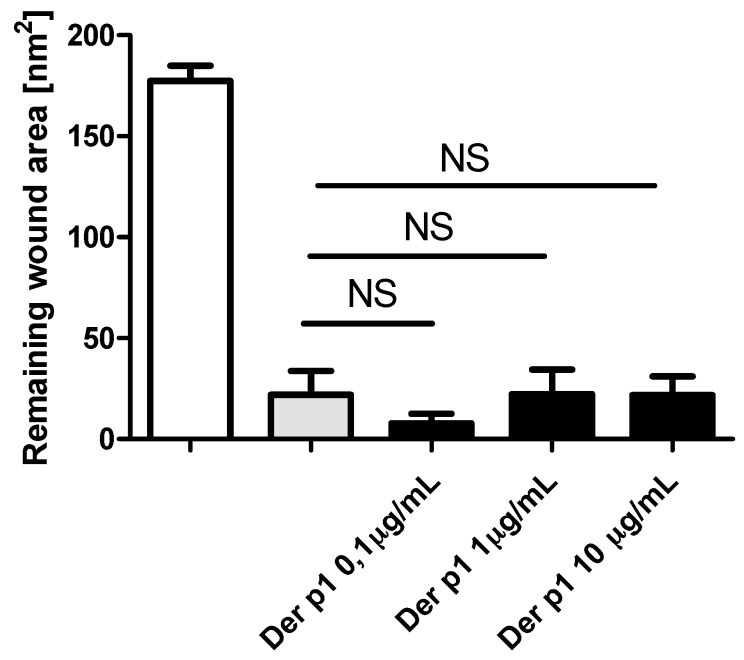
Effect of Der p1 on wound repair in BEAS-2B (*n* = 6 for 1 μg/mL, *n* = 4 for 0.1 and 10 μg/mL), 24 h post injury. NS: no statistically significant difference.

Incubation of wounded cells with Der p1 did not affect epithelial cell regeneration in our model (Figure 4).

### 2.4. Preparation of Conditioned Media from RV1b- and PIV3-Infected Cells

BEAS-2B cells cultured into confluence were placed in medium without serum or additives and then infected with either RV1b or PIV3 at a multiplicity of infection 0.1 as described before [22,23,24]. After 1 h of incubation, the culture medium was replaced by fresh minimal Eagle medium supplemented with 2% Fetal Calf Serum. The supernatants from infected and not-infected cells were collected at 24, 48, and 72 h post infection and used in further experiments.

### 2.5. Effect of Conditioned Media from RV1b-Infected Epithelial Cells (ECs) on Wound Repair

**Figure 5:**
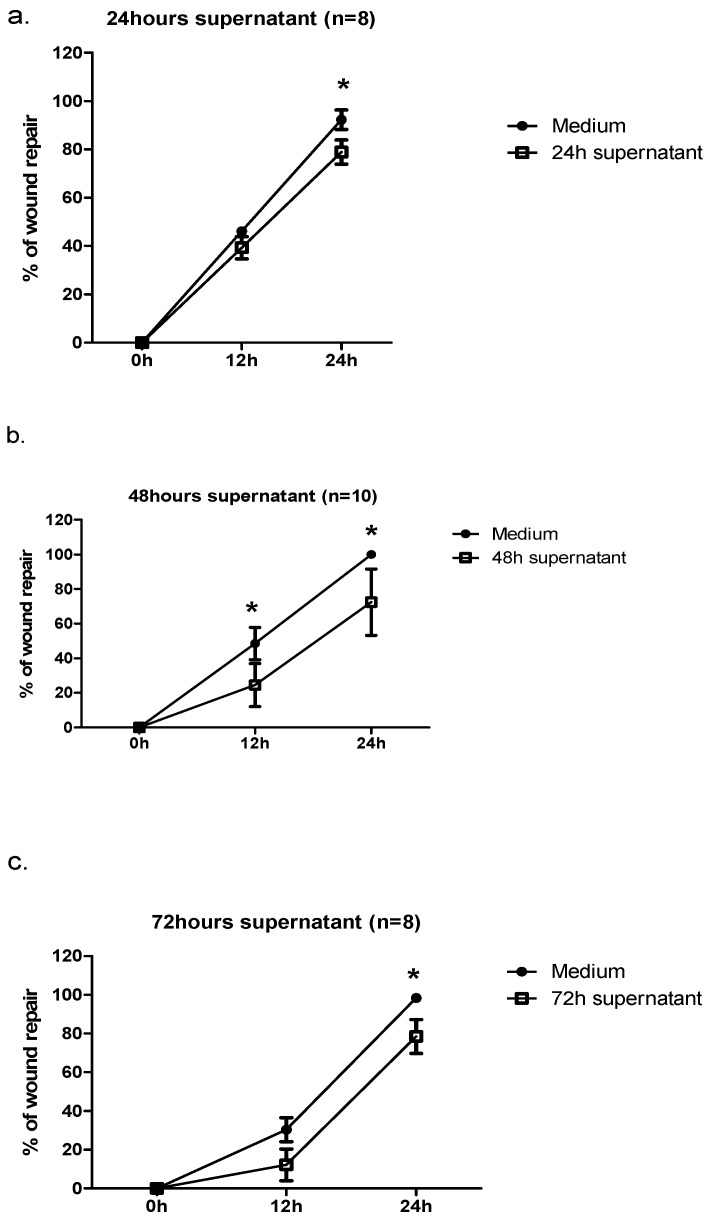
Effect of supernatant from RV1b-infected epithelial cells on wound repair. (**a**) 24 h supernatant; (**b**) 48 h supernatant; (**c**) 72 h supernatant; * *p* < 0.05.

Incubation of bronchial epithelial cells with supernatants from RV1b-infected cells decreased repair response 24 h post injury. In experiments with supernatants collected 24 h post infection, we observed 75% regeneration versus 92% in controls. Addition of 48 h and 72 h supernatants to epithelial cells also decreased wound repair by 75% versus 100% and 82% versus 98%, respectively, 24 h post injury. Moreover, for the 48 h and 72 h supernatants we observed the difference in wound repair 12 h post injury (Figure 5a–c).

We compared repair in cultures stimulated with supernatant from infected cells to cultures stimulated with supernatants from not-infected cells.

Recorded differences in wound area 24 h post injury are presented as mean wound area at different time points in Table 1a,b.

Addition of supernatants from cells infected with another respiratory virus—human parainfluenza virus type 3 (PIV3)—also inhibited epithelial repair in BEAS-2B cell cultures to a similar degree (71%, 55%, and 72% for 24 h, 48 h, and 72 h supernatants versus 100%, 95%, and 100% in cultures with media from not-infected cells, respectively).

To investigate whether LPS-induced repair inhibition is caused by TLR stimulation cells were incubated with the TLR4 inhibitor LPS-RS before addition of LPS.

**Figure 6:**
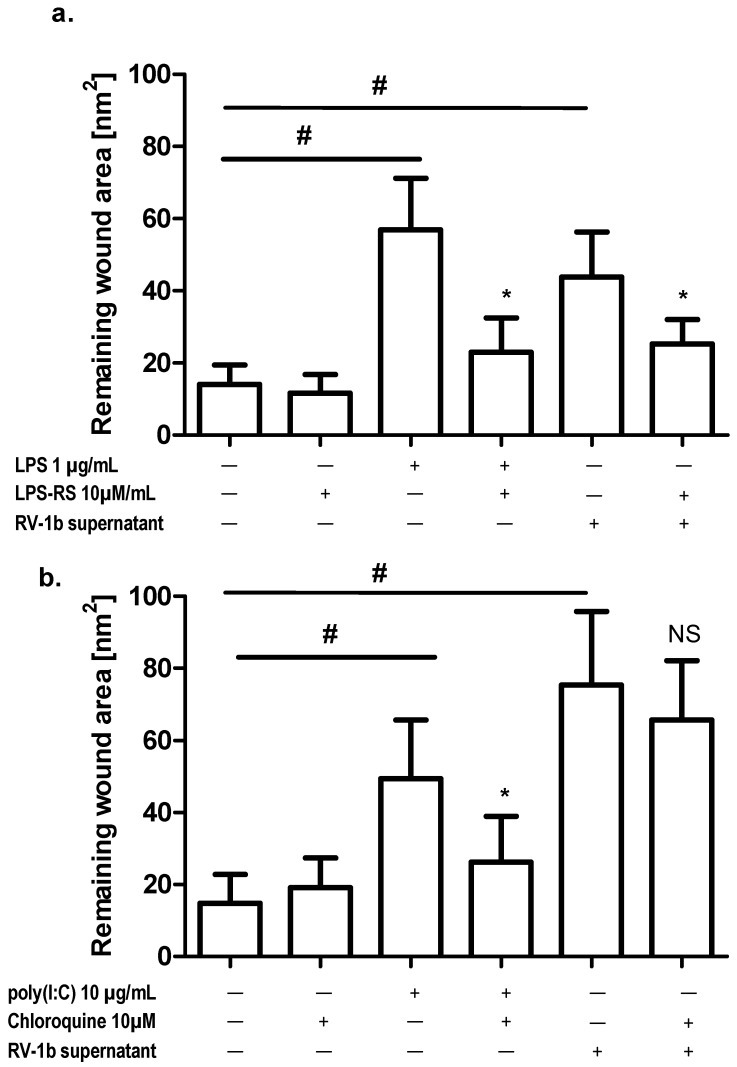
Effect of TLR4 and TLR3 inhibitors on wound repair; (**a**) TLR4 inhibitor LPS-RS; (**b**) TLR3 inhibitor chloroquine. * *p* < 0.05; # *p* < 0.05; NS: no statistically significant difference.

Preincubation of injured and not-injured cells with LPS-RS decreased LPS-induced repair inhibition. This effect was also seen in cultures stimulated by conditioned media from RV1b-infected cells (Figure 6a). An additional set of experiments with chloroquine, a TLR3 inhibitor, also revealed an inhibitory effect on poly(I:C)-induced epithelial cells impaired repair response, but there was no effect on conditioned-media-induced repair inhibition (Figure 6b).

**Figure 7:**
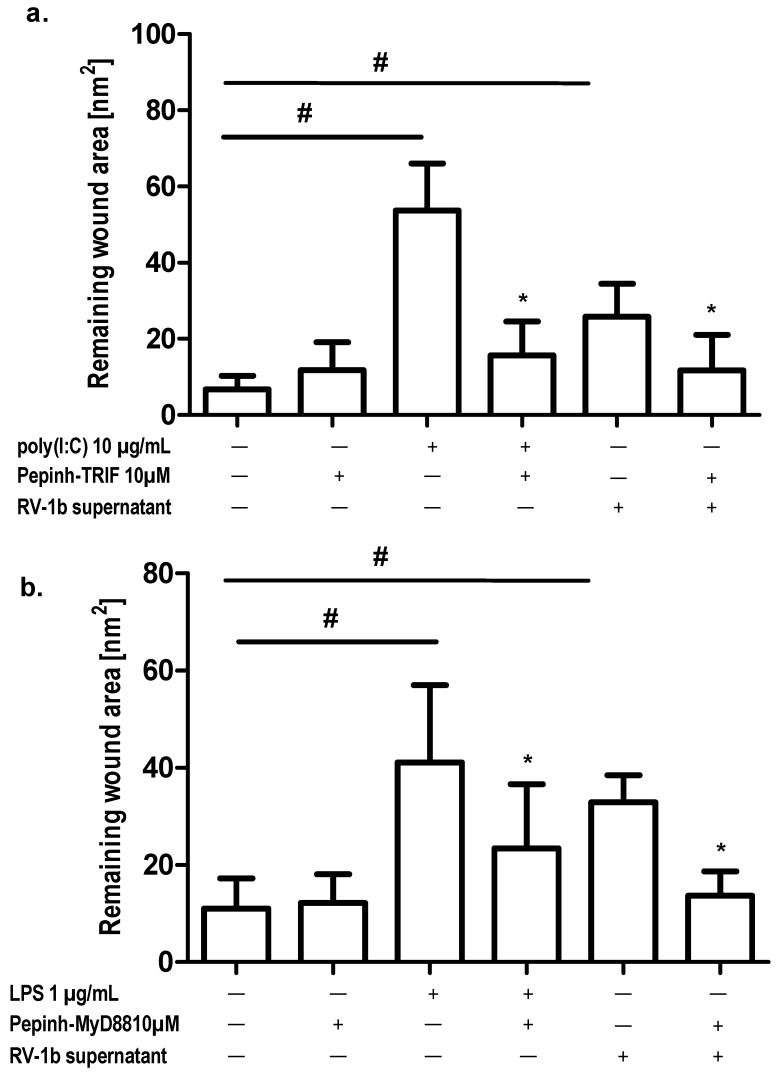
Effect of TLR intracellular pathway inhibitors on wound repair; (**a**) TRIF inhibitory peptide Pepinh-TRIF; (**b**) MyD88 inhibitory peptide Pepinh-MyD88. *p < 0.05; # *p* < 0.05.

Next, we evaluated whether the addition of TLR intracellular pathway inhibitors modulated epithelial cells regeneration. Addition to wounded and not-wounded cultures of Pepinh-TRIF (a TRIF inhibitory peptide) and Pepinh-MyD88 (a MyD88 inhibitory peptide) significantly decreased the inhibitory effects of LPS and poly(I:C) on wound regeneration. These effects were also observed in cultures stimulated with conditioned media (Figure 7a,b).

### 2.6. Immune Response in Injured and Not-Injured Airway Epithelial Cells

#### Cytokine mRNA Expression and Protein Release in Bronchial Epithelial Cells in Response to Injury

**Figure 8:**
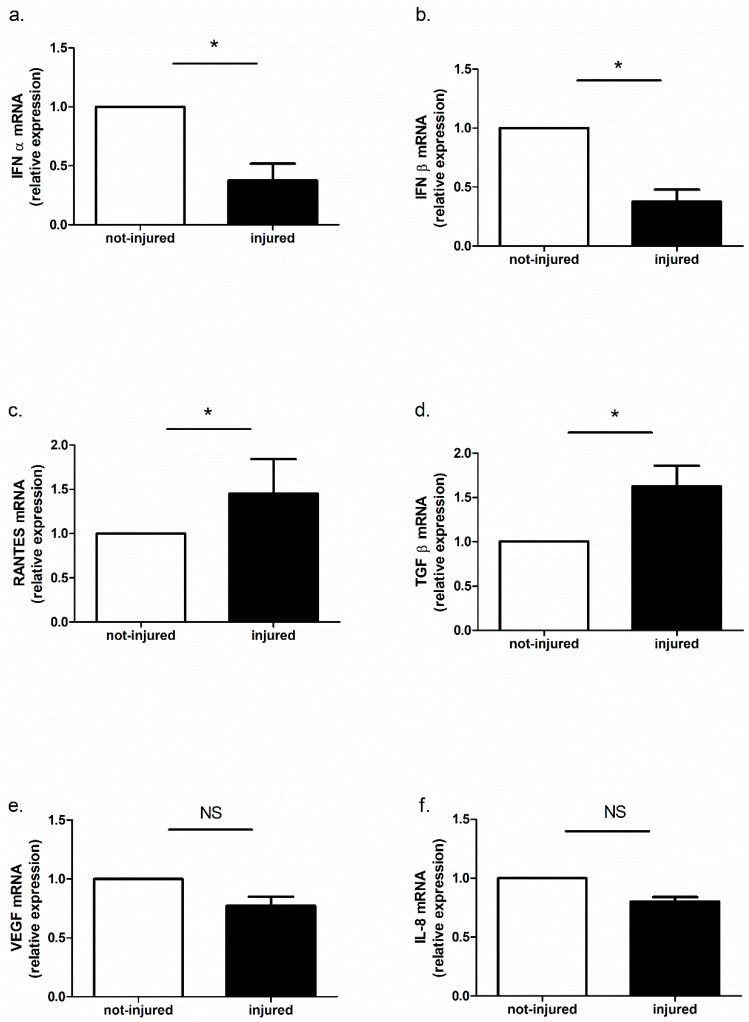
Cytokines mRNA expression in wounded and not-wounded epithelial cells. (**a**) IFN-α; (**b**) IFN-β; (**c**) RANTES; (**d**) TGF-β; (**e**) VEGF; (**f**) IL-8; * *p* < 0.05; NS: no statistically significant difference.

In our model, we observed IFN-α, IFN-β, TGFβ, VEGF, RANTES, and IL-8 mRNA expression. TGF-β and RANTES mRNA expression was higher in injured epithelial cells and IFN-α and IFN-β mRNA expression was lower in damaged epithelium as compared to not-injured epithelium. There were no differences in IL-8 and VEGF expression between wounded and not-wounded cell cultures (Figure 8a–f).

LPS stimulation decreased IFN-α and IFN-β mRNA expression in both not-injured and injured BEAS-2B cell cultures. LPS did not affect RANTES, TGF-β, VEGF, and IL-8 mRNA expression either in wounded or in not-wounded cells.

Addition of poly(I:C) to wounded and not-wounded epithelial cells causes a decrease in IFN-α and IFN-β in not-injured and an increase in injured epithelial cells. In injured epithelial cells, a decrease in TGF-β was also observed and there was no effect of poly(I:C) on TGF-β mRNA expression in not-injured cells. Stimulation with poly(I:C) did not affect RANTES, VEGF, and IL-8 expression.

**Figure 9:**
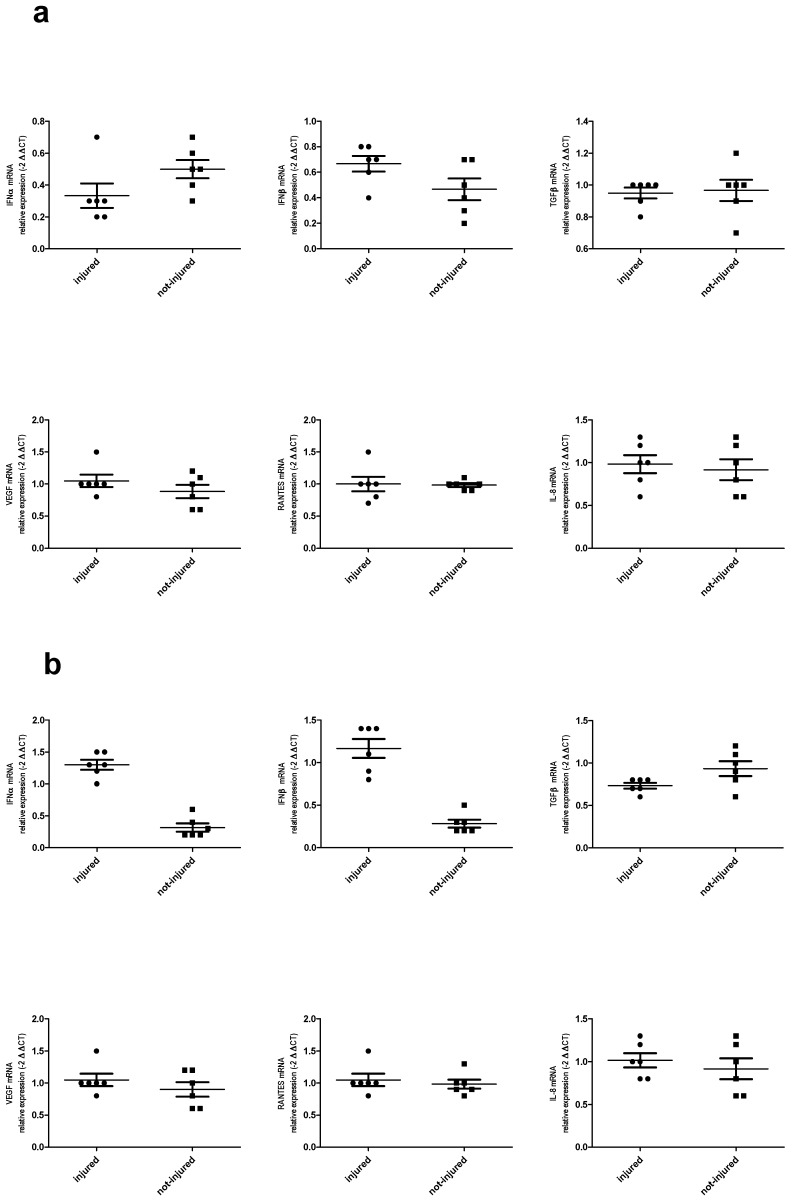
LPS—(**a**) and poly (I:C)—(**b**) induced cytokine expression in injured and not-injured cells.

The effect of LPS and poly(I:C) on cytokine expression in wounded and not-wounded cells is presented in Table 2 and Figure 9.

Out of the six cytokines measured in BEAS-2B supernatants, we found measurable concentrations of IFN-α, IFN-β, RANTES, TGFβ, VEGF, and IL-8.

**Figure 10:**
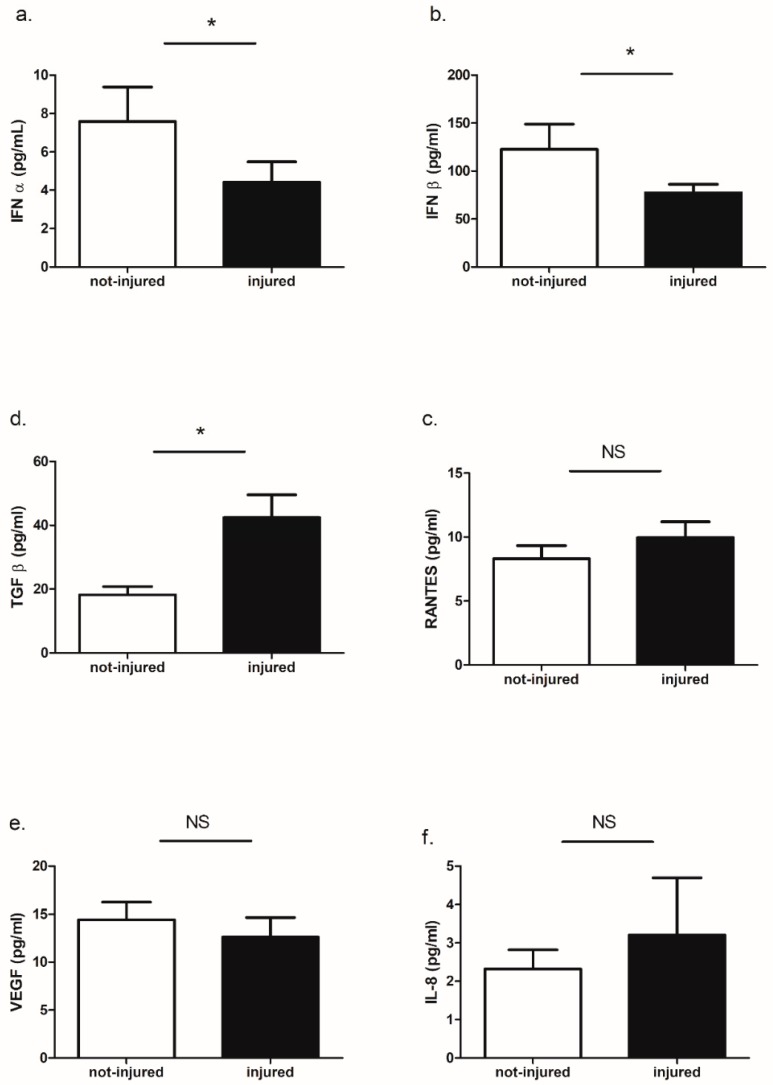
Cytokines protein release in wounded and not-wounded epithelial cells. (**a**) IFN-α; (**b**) IFN-β; (**c**) RANTES; (**d**) TGF-β; (**e**) VEGF; (**f**) IL-8; * *p* < 0.05; NS: no statistically significant difference.

We observed a higher concentration of TGFβ in wounded as compared to not-wounded cells and a lower concentration of IFN-α and IFN-β in wounded cells. There was no influence of injury to RANTES and IL-8. Surprisingly, there was also no effect on VEGF release (Figure 10a–f).

## 3. Discussion

In this study, we have documented for the first time that toll-like receptors are involved in the modulation of airway epithelial cells regeneration. To investigate the regeneration, a previously described and validated [25] experimental model of human epithelial cell monolayers was used. The wound repair and the immune responses were assessed in epithelial cells by a scratch assay and by comparing mediators released by injured and not-injured cells. The human epithelial cell line (BEAS-2B) used in this study [26,27] has been previously used as an in vitro model of wound injury [28] and has been shown to release inflammatory mediators and express relevant intracellular signaling pathways.

TLRs play an important role in the induction of innate immune responses to infections. Thus, we hypothesized that these receptors may modulate regeneration of epithelial cells during respiratory infection [29]. To assess the role of TLR stimulation, we used the TLR3 agonist poly(I:C) and the TLR4 agonist LPS. After addition of poly(I:C) to mechanically damaged monolayers of BEAS-2B cells, a marked inhibition of wound repair was observed. The inhibitory effect of poly(I:C) was dose-dependent, and it was abolished when chloroquine, a well-known TLR3 signaling inhibitor [30], was added to cell cultures, confirming the specific TLR3-driven mechanism of cell regeneration inhibition. Furthermore, addition of a TRIF inhibitory peptide significantly decreased the inhibitory effects of poly(I:C) on wound regeneration, suggesting involvement in this process of a specific intracellular pathway. Since TLR3 recognizes double-stranded (ds)RNA, which is the nuclear material of many viruses [31] our data suggest that inhibition of epithelial cell regeneration may be an important mechanism associated with injury of epithelial cells observed during viral infection in the airways of asthma patients [24].

Addition of LPS to damaged epithelial cells also resulted in a dose-dependent decrease in epithelial monolayers repair, and this inhibitory effect was blocked by preincubation of cells with a TLR4 receptor inhibitor (LPS-RS). Furthermore, inhibition of the TLR4 signaling adaptor MyD88 showed a similar effect to that of the LPS inhibitor. Since TLR4 is activated by LPS and other bacterial products [32], it is tempting to speculate that bacterial infections may, similarly to viruses, modulate airway epithelial cell repair occurring in response to injury induced by environmental factors.

Previous studies have documented airway epithelial barrier disruption by different factors, such as cigarette smoke [33,34], allergens [35], and viral infection [10,13]. However, there are only two studies referring to the role of viral infection in epithelial regeneration [14,15], and ours is the first one describing the involvement of toll-like receptors in inhibition of airway epithelial repair.

It has been shown previously that the mite allergen Der p1 is involved in allergic airway inflammations able to damage the epithelial cell barrier by disrupting the tight junction directly through protease activation and indirectly through protease-activated receptor-2 (PAR2) [35,36]. These effects result from a TLR4-dependent activation of NF-κB by house dust mites. In our study, we asked the question of whether an allergen may impair regeneration of the epithelium and we observed that incubation of wounded cells with Der p1 did not directly affect epithelial cell regeneration. These data suggest that the effect of TLR stimulation on repair response may depend on the influence of other factors, such as cytokines and different inflammatory mediators.

Having in mind the important role of respiratory viruses in airway epithelial cell damage, we attempted to assess the indirect effect of respiratory viral infection on epithelial regeneration. Incubation of bronchial epithelial cells with supernatants collected over 72 h from either RV1b- or PIV3-infected cells decreased the repair process 24 h post injury. Preincubation of injured and not-injured cells with the LPS inhibitor decreased supernatant-induced repair inhibition. This effect was also seen in cultures stimulated by conditioned media from RV1b in the presence of either TRIF inhibitor or MyD88 inhibitor. These findings may suggest that supernatants activated TLRs by danger-associated molecular patterns (DAMPs) and are consistent with data suggesting that respiratory epithelial cells respond to infection and produce endogenous DAMPs, such as for example ATP, HMGB1, and S100 proteins [37,38]. DAMPs activate epithelial cell-intrinsic pattern recognition pathways and also recruit and activate cells of the immune system [39].

Our study documented also the presence of an ongoing immune response in mechanically wounded epithelial cells. In our model, we noticed that TGF-β and RANTES mRNA expression was higher in injured epithelial cells while IFN-α and IFN-β mRNA expression was lower in damaged epithelium as compared to not-injured. These results are consistent with previous studies showing that epithelial cells produced TGF-β and RANTES [40,41] and that injury is a sufficient stimulus to trigger and maintain inflammation in the autocrine pathway. It is not clear why the expression of interferon type I was lower in injured cells. It is possible that injury caused an imbalance between transcriptional factors resulting in decreased expression of some genes. It was also interesting that stimulation of wounded and not-wounded epithelial cells with poly(I:C) caused a decrease in IFN-α and IFN-β in not-injured and an increase in injured epithelial cells. An increase of IFN-β in BEAS-2B cells stimulated with poly(I:C) and infected by Influenza A virus has been previously shown [42]. Our observation underlines evidence that injury has an additional effect on immune response induced by pathogens and that TLR-mediated signaling responses are differentially regulated in wounded and not-wounded cells.

In summary, our study demonstrated that regeneration of the airway epithelium is modulated by microbial products via toll-like receptors and has obtained some insights into the mechanism of loss of epithelial barrier integrity observed during various respiratory infections. Furthermore, our data suggest that modulation of TLR activity may be potentially used for influencing airway epithelial regeneration.

## 4. Materials and Methods

### 4.1. Cell Culture

Human bronchial epithelial cell line BEAS-2B was obtained from the American Type Culture Collection (ATCC, Manassas, VA, USA) and was cultured in Eagle’s minimal essential medium with Earle’s salt (Sigma-Aldrich, Munich, Germany), buffered with NaHCO_3_, and supplemented with 10% (*v*/*v*) fetal bovine serum (FBS, Invitrogen, Life Technologies, Carlsbad, CA, USA) and 40 µg/mL of gentamycin (Sigma-Aldrich) in a humified 5% CO_2_ incubator. Confluent cultures were trypsynized with 0.1% trypsin-EDTA solution (Sigma-Aldrich) and cells were seeded on uncoated 35-mm-diameter Petri dishes with 2-mm square grids on the bottom surfaces (Invitrogen, Life Technologies) for further experiments. An MTT assay was used for the cell viability assessment.

### 4.2. Wound Repair Assay

For assessment of cell regeneration, confluent cultures were mechanically scratched with a 10-μL pipette tip and washed with serum-free culture medium to remove damaged cells. Next, digital photos of identifiable squares of impaired cultures were taken at 0, 12 h, and 24 h after injury using an Olympus 3040Z digital camera with an Olympus CK2 inverted microscope (Olympus Optical, Middlesex, UK). The area of unpopulated cells was measured with image analysis software (CellSens Standard, Olympus). In each experiment, four squares on each plate were analysed the mean was calculated and each experiment was repeated at least three times.

In preliminary experiments, the wound area was analysed every 3 h for 24 h to assess the time and rate of repair, and in subsequent experiments the damaged area 12 h and 24 h post injury was compared with the initial wound area. Wound repair (WR) was expressed as a percentage of the initial wound area measured at the start of the experiment and was calculated using the following formula: %WR = ((initial wound area − 24 h wound area)/initial wound area) × 100.

### 4.3. Viability Assay

Cell viability was assessed by an MTT assay (Sigma-Aldrich). At specified time points, the culture media was removed, and epithelial cells were incubated with 0.5 mg/mL MTT (thiazolyl blue, 3-(4,5-dimethylthiazol-2-yl)-2,5-diphenyltertrazolium bromide) solution in an incubator at 37 °C for 3 h. After removing the medium, DMSO was added to solubilize the blue-colored tetrazolium and the plates were then shaken for 5 min. Two hundred microliters (200 μL) of MTT solution were transferred to a 96-well plate. The absorbance at 570 nm was monitored. Data were expressed as a percentage of the optical density (OD) at 570 nm of a wounded cells sample compared to unwounded cells.

### 4.4. Cell Stimulation and Treatment of ECs with Conditioned Media from Infected Cells

Damaged and not-damaged cultures (controls) were stimulated with the TLR agonists poly(I:C) (Sigma-Aldrich) (0.1 µg/mL, 1 µg/mL, 10 µg/mL) and LPS (Sigma-Aldrich) (1 µg/mL, 10 µg/mL, 50 µg/mL, 100 µg/mL) for 60 min; the allergen Der p1 (Indoor Biotechnologies Ltd., Cardiff, UK); and conditioned media from virus-infected epithelial cells. TLR agonists and conditioned media were added either alone or in combination with the TLR inhibitor LPS-RS (Sigma-Aldrich) or the TLR inhibitor chloroquine (Sigma-Aldrich) or the intracellular pathway inhibitor Pepinh-TRIF (InvivoGen, Toulouse, France) or the intracellular pathway inhibitor Pepinh-MyD88 (InvivoGen).

### 4.5. Preparation of Conditioned Media from RV1b-Infected Cells

Confluent BEAS-2B cell cultures were infected with RV1b at 0.1 MOI. After 1 h of incubation with RV1b, supernatants were removed and the cells were cultured in MEM supplemented with 2% FCS for 24, 48, and 72 h. At these three time points, supernatants were collected and prepared for further experiments by centrifugation, filtration, and inactivation with UV irradiation for 30 min.

### 4.6. Measurement of Cytokines and Growth Factors

IFN-α, IFN-β, TGF-β, VEGF, RANTES, and IL-8 levels were measured in cell supernatants by commercially available ELISA kits (R&D Systems, Minneapolis, MN, USA) according to the manufacturer’s specifications.

The sensitivity of the immunoassays was as follows: IFN-α 12.5 pg/mL, IFN-β 50 pg/mL, TGF-β 9.87 pg/mL, RANTES 3 pg/mL, IL-8 1.5 pg/mL.

### 4.7. RNA Isolation and Quantitative Polymerase Chain Reaction (PCR)

Total RNA was extracted from epithelial cells using the RNeasy Mini Kit (Qiagen, Hilden, Germany) according to the manufacturer’s instructions. One microgram (1 µg) of total RNA was reverse-transcribed into cDNA by using a RevertAid H Minus First Strand cDNA Synthesis Kit (Thermo Scientific, Vienna, Austria). TaqMan probes specific for IFNA1, IFNB1, RANTES, TGFβ, VEGFA, IL-8, and GAPDH (as a reference gene), were purchased from Life Technologies. Gene expression analysis was performed on a Step One Plus™ Real-Time PCR System (Applied Biosystems, Carlsbad, CA, USA). The mRNA expression was assessed using the relative quantification method (2^−ΔΔ*C*t^ method).

### 4.8. Measurement of Mediators

IFN-α, IFN-β, TGF- β, VEGF, IL-8, and RANTES levels were measured in supernatants from injured and not-injured cells by commercially available ELISA kits (R&D Systems, Minneapolis, MN, USA) according to the manufacturer’s specifications. The sensitivity of the immunoassays was as follows: IFN-α 12.5 pg/mL, IFN-β 50 pg/mL, RANTES 3 pg/mL, IL-8 1.5 pg/mL, TGF-β 15.4 pg/mL, VEGF 9 pg/mL.

### 4.9. Statistical Data Analysis

Statistical analysis was performed with Statistica version 10 PL (Statsoft Polska, Krakow, Poland). Data are presented as means and standard errors of the means. Gene expression and cytokine concentrations were analysed using Kruskal–Wallis analysis of variance (ANOVA) followed by Wilcoxon’s ranked-pairs for paired data. When appropriate, a Mann–Whitney U-test was performed and a subsequent post hoc analysis was done using the Bonferroni-adjusted α-method. A *p* value lower than *0.05* was considered statistically significant.

## Figures and Tables

**Table 1:** (**a**) Mean wound area in cultures stimulated with the TLR agonists LPS and poly(I:C); (**b**) Mean wound area in cultures stimulated with supernatant from infected epithelial cells.

(**a**)				
**Time Post Injury**	**0 h**		**24 h**	
Mean wound area (nm^2^)	control	LPS 1 μg/mL	control	LPS 1 μg/mL
	**143.2 ± 13.8**	**151.8 ± 9.9**		**22.1 ± 7.3**
		LPS 10 μg/mL		LPS 10 μg/mL
		**150.2 ± 14.9**	**14.1 ± 4.7**	**56.8 ± 14.3**
		LPS 50 μg/mL		LPS 50 μg/mL
		**137.2 ± 16.1**		**69.2 ± 12.2**
**Time Post Injury**	**0 h**		**24 h**	
Mean wound area (nm^2^)	control	poly(I:C) 0.1 μg/mL	control	poly(I:C) 0.1 μg/mL
	**133.6 ± 13.1**	**151.8 ± 9.9**		**7.7 ± 4.9**
		poly(I:C) 1 μg/mL	**3.7 ± 2.6**	poly(I:C) 1 μg/ml
		**150.2 ± 14.9**		**33.1 ± 3.5**
		poly(I:C) 10 μg/mL		poly(I:C) 10 μg/ml
		**137.2 ± 16.1**		**73.1 ± 4.8**
(**b**)				
**Time Post Injury**	**0 h**		**24 h**	
Mean wound area (nm^2^)	control	RV1b 24 h supernatant	control	RV1b 24 h supernatant
	**163.4 ± 14.5**	**143.7 ± 12.7**	**17.4 ± 6.7**	**33.8 ± 18.1**
	control	RV1b 48 h supernatant	control	RV1b 48 h supernatant
	**167.9 ± 13.6**	**150.2 ± 14.9**	**1.1 ± 0.8**	**44.6 ± 23.9**
	control	RV1b 72 h supernatant	control	RV1b 72 h supernatant
	**146.9 ± 19.1**	**137.2 ± 16.1**	**1.1 ± 0.7**	**23.1 ± 11.6**
Mean wound area (nm^2^)	control	PIV3 24 h supernatant	control	PIV3 24 h supernatant
	**168.0 ± 18.6**	**151.0 ± 18.5**	**0**	**66.6 ± 36.8**
	control	PIV3 48 h supernatant	control	PIV3 48 h supernatant
	**120.2 ± 12.7**	**147.7 ± 13.3**	**11.6 ± 8.7**	**73.9 ± 32.1**
	control	PIV3 72 h supernatant	control	PIV3 72 h supernatant
	**120.8 ± 13.7**	**137.0 ± 15.5**	**0**	**90.2 ± 35.3**

**Table 2:** Comparison of cytokine expression in injured and not-injured cells with and without stimulation with LPS and poly(I:C). NS, no statistically significant difference.

	Injured versus Not-Injured	LPS		poly(I:C)	
		Not-Injured	Injured	Not-Injured	Injured
IFN-α	↓	↓	↓	↓	↑
IFN-β	↓	↓	↓	↓	↑
TGF-β	↑	NS	NS	NS	↑
VEGF	↓	NS	NS	NS	NS
RANTES	↑	NS	NS	NS	NS
IL-8	↓	NS	NS	NS	NS

## Abbreviations

ECs	epithelial cells
LPS	lipopolysaccharide
TLRs	toll-like receptors
RV1b	rhinovirus type 1B
PRRs	pathogen recognition receptors
